# Signal transmission through elements of the cytoskeleton form an optimized information network in eukaryotic cells

**DOI:** 10.1038/s41598-019-42343-2

**Published:** 2019-04-16

**Authors:** B. R. Frieden, R. A. Gatenby

**Affiliations:** 1 College of Optical Science, University of Arizona, Tucson, AZ USA; 2 Department of Integrated Mathematical Biology, Moffitt Cancer Center, Tampa, FL USA

**Keywords:** Nanoscale biophysics, Cell signalling

## Abstract

Multiple prior empirical and theoretical studies have demonstrated wire-like flow of electrons and ions along elements of the cytoskeleton but this has never been linked to a biological function. Here we propose that eukaryotes use this mode of signal transmission to convey spatial and temporal environmental information from the cell membrane to the nucleus. The cell membrane, as the interface between intra- and extra-cellular environments, is the site at which much external information is received. Prior studies have demonstrated that transmembrane ion gradients permit information acquisition when an environmental signal interacts with specialized protein gates in membrane ion channels and producing specific ions to flow into or out of the cell along concentration gradients. The resulting localized change in cytoplasmic ion concentrations and charge density can alter location and enzymatic function of peripheral membrane proteins. This allows the cell to process the information and rapidly deploy a local response. Here we investigate transmission of information received and processed in and around the cell membrane by elements of the cytoskeleton to the nucleus to alter gene expression. We demonstrate signal transmission by ion flow along the cytoskeleton is highly optimized. In particular, microtubules, with diameters of about 30 nm, carry coarse-grained Shannon information to the centrosome adjacent to the nucleus with minimum loss of input source information. And, microfilaments, with diameters of about 4 nm, transmit maximum Fisher (fine-grained) information to protein complexes in the nuclear membrane. These previously unrecognized information dynamics allow continuous integration of spatial and temporal environmental signals with inherited information in the genome.

## Introduction

Survival and proliferation of living systems require them to continuously acquire, process, and respond to information^1^ from the environment for threats, opportunities, or (in the case of multicellular organisms) instructions from local tissue^2^. The cell membrane, as the interface between a cell and its environment, is the site at which much of this environmental information is received. Some environmental changes, such as perturbations in osmolarity, temperature and pH, typically affect all regions of the cell membrane equally and simultaneously. Other information, such as (1) the presence of a potential predator or food source; or, (2) in the tissue of a highly ordered multicellular organism, the space-time location of a target cell must be spatially and temporally resolved.

**Figure 1:**
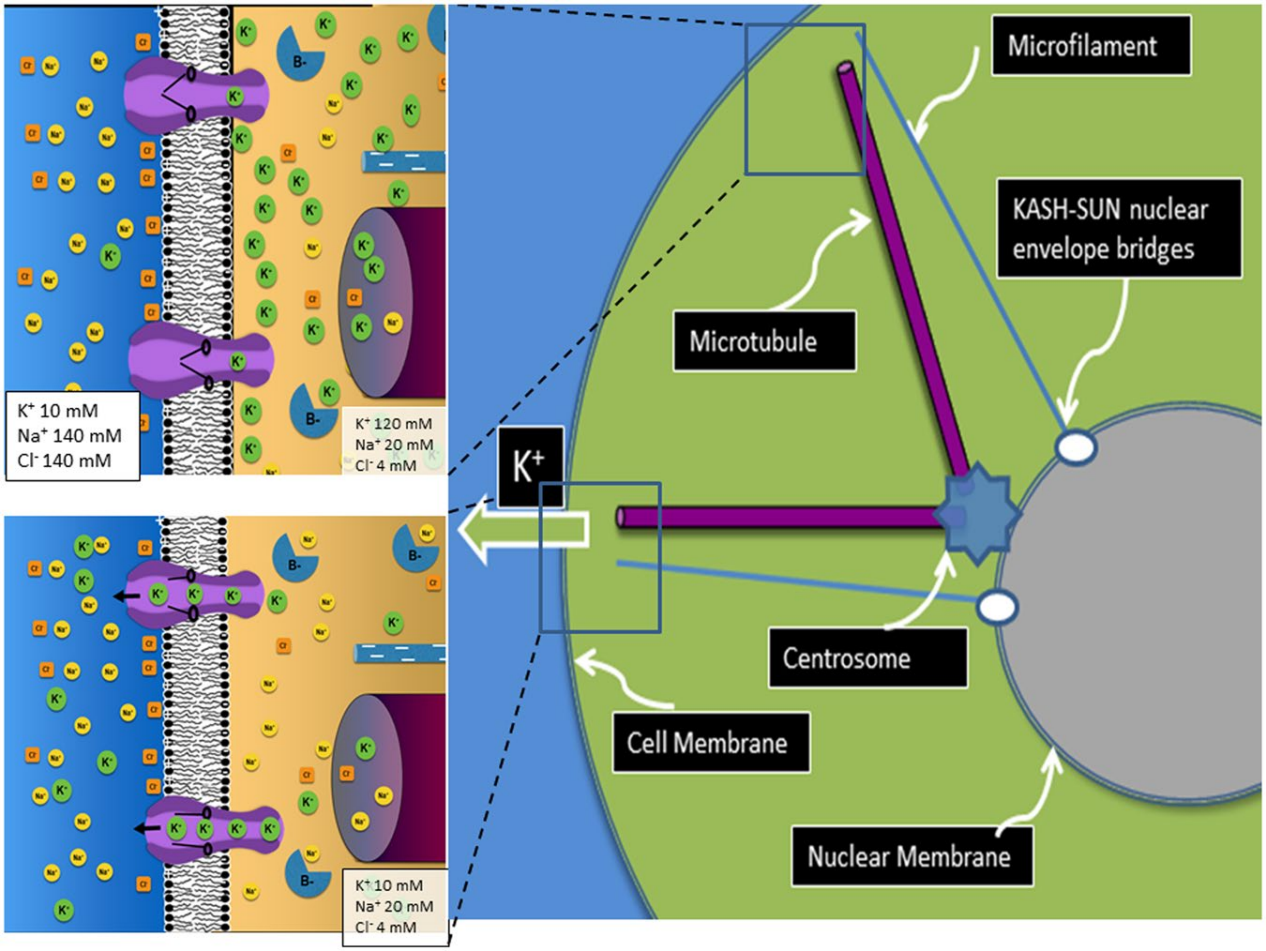
Information dynamics in and around the cell membrane and transmission to central organelles. The resting state of the membrane, with large transmembrane concentration gradients of K+, Na+ and Cl− is shown in the upper left panel. In the lower left panel, an environmental signal causes the gates in transmembrane K+ channels to open. This allows rapid flow of K+ out of the cell briefly altering the ion concentrations and charge balance in the cytoplasm. Prior studies have shown these ion dynamics alter localization and function of peripheral membrane proteins permitting analysis of and response to the environmental perturbation. This ion flux in the cytoplasm adjacent to the cell membrane can also enter the channel of adjacent microtubules which allows transmission of coarse-grained information to the centrosome (see Fig. 3). The ion flux can also change the electrical potential at the distal end of a microfilament allowing electron flow along the wire-like structure transmitting (Fig. 3) fine-grained information to a protein complex in the nuclear membrane which can alter gene expression and chromosomal location.

A prior study^3^ demonstrated that the steep transmembrane ion gradients in eukaryotes are critical for receiving and processing environmental information. Information is received when some perturbation causes the protein gates in transmembrane ion channels to open. The subsequent flow of one or more ions into or out of the cell along these pre-existing electro-chemical gradients can induce local changes (Fig. 1) that promote an adaptive (both fast and targeted) cellular response. For example, an outflow of K^+^ (the dominant mobile cation in the cytoplasm) may reduce the shielding of fixed negative charges on the inner leaf of the cell membrane, enhancing electrostatic forces for attracting or repelling charges on nearby macromolecules. Furthermore, the activity of many enzymes is dependent on cation concentrations, so that a local fluctuation may increase or decrease their activity^4^.

The role of transmembrane ion movement has been recognized for decades as the mechanism of nerve conduction^5^. As expressed by the Hodgkin-Huxley (H-H) equations^6^, propagating depolarization waves along an axon are generated by sequential transmembrane flows of ions through membrane channels. There is one H-H equation for each ion. Our model proposes^3^ that the ion dynamics that produce a traveling depolarization wave in neurons are, in fact, a specialized application of membrane information dynamics that are universally obeyed by eukaryotes.

Here we address the question of how environmental information that is transmitted through the cell membrane through ion fluxes is communicated internally to other components of the cell. We expect that many environmental perturbations (e.g. a localized mechanical deformation by a small environmental object) may only elicit and require a local response. However, some signals received at the membrane, because of their content, amplitude, or spatiotemporal frequency, may require a global (or ‘coordinated’) cellular response including increased energy production in the mitochondria^7,8^ and changes in gene expression^9^ or translation within the nucleus and endoplasmic reticulum^10^.

**Figure 2:**
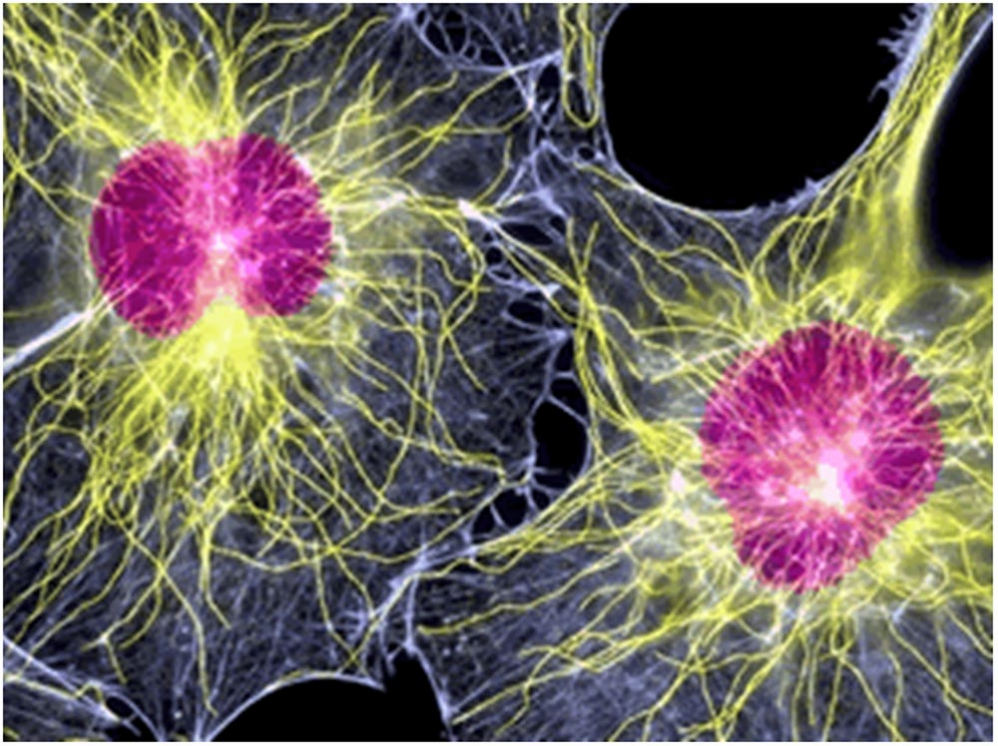
Distribution of microfilaments and microtubules in eukaryotes. Immunohistochemistry stains showing the distribution of microtubules (green) and microfilaments (red) within normal fibroblasts.

**Figure 3:**
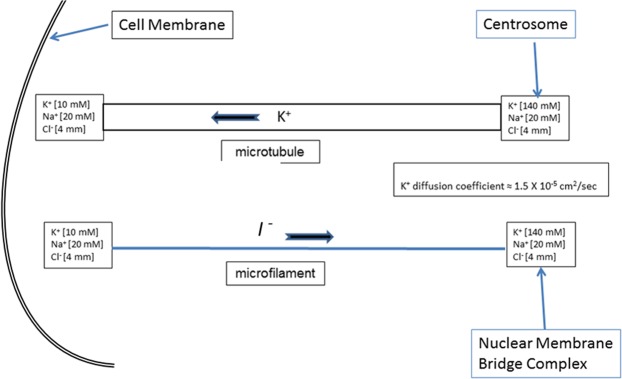
Cytoskeletal structures as information conduits. An environmental perturbation that causes transmembrane flow of K+ out of the adjacent cytoplasm (Fig. 2) generates a transient ion gradient along the length of the hollow core of a microtubule or a potential gradient along the microfilament which forms a wire-like conductor for ion flow.

We propose that information encoded in local fluxes of ions in the cytoplasm adjacent to the cell membrane can be transmitted to other organelles by elements of the intracellular cytoskeleton, its microtubules and microfilaments (Figs 2 and 3). These form organized networks throughout eukaryotic cells that are often complex and dynamic^11^ (Fig. 2).

Although the elements of the cytoskeleton are primarily involved in cellular shape and movement^8^, they can also serve as both biomechanical and electrical conduits of information that can alter gene expression^9^ and chromosomal location^9,12^. The ability of microfilaments and microtubules to conduct electrons and ions has been extensively documented^13^. Microfilaments are actin polymers which maintain high levels of negative surface charges permitting highly dynamical interactions with both within the microfilament and in exchange with cytoplasmic counterions^14,15^. A number of studies have demonstrated charge centers with corresponding counter ion clouds along the microfilament axis permitting ionic waves propagating along its long axis^15–18^. This conductance takes on a specific form as Cantiello *et al*. demonstrated actin filaments propagate electrical signal via soliton waves^18^ so that the signal is virtually lossless. Ionic conduction along the length of microtubules has also been observed and S. The precise mechanism is not clear^19^ but may include diffusion along the central channel^20^ and ion redistribution along the microtubule as a result of variations in cation (Na, K, Ca) flux through nanopores along the microtubule wall^21^. Furthermore, microtubules are capable of amplifying ionic signal waves^22^. There is also experimental evidence that microtubules can regulate VDAC ion channels in the mitochondria^23^ and that microtubules can influence^24^ and be influenced by the extracellular matrix^25^ (ECM), so that there is a dynamic and ongoing exchange of information between the cytoskeleton and cell exterior. Finally, recent work by Santelices *et al*.^26^ has experimentally demonstrated that microtubule responses to AC electric signals are frequency and ion concentration dependent.

The cytoskeleton fibers are often arrayed in organized patterns along the radius of the cell from the nuclear membrane to the cell membrane (Fig. 1). Furthermore, the proximal ends of microtubules, which typically join together in the centrosome^27^ adjacent to the nuclear membrane and microfilaments, are often bound to multiprotein structures (e.g. the KASH-SUN complex^28^**)** in the outer nuclear membrane. Molecular tethers are, thereby, formed that have diverse functions, e.g. gene transcription, as well as transmission of forces for chromosome movements and nuclear migration. Furthermore, actin filaments directly alter nuclear pore ion channel activity, thus altering the ionic milieu of the nucleoplasm^29^.

Hence we investigate the potential of microtubules and microfilaments to act as information conductors that link the cell membrane with central cellular structures including the nucleus, mitochondria and endoplasmic reticulum. These thereby form a distributed information network of conductors.

## Summary of the Biological Model

Our biological information model proposes environmental perturbations can be detected by specialized gates on membrane ion channels. When the gate opens, ions specific to the channel will flow along concentration gradients into or out of the cell. Depending on the number of open channels, and the duration of that open state, the cytoplasm adjacent to the channels will undergo a rapid change in ion concentrations, charge density, and osmolarity. Once the gates are closed, rapid re-equilibration will occur through diffusion from adjacent regions of the cytoplasm and activation of transmembrane ion pumps.

When the transmembrane ion flows within one or a few channels (measured to be about 10^5^ ions/second per channel) are very brief, such as depicted by the Hodgkin-Huxley (H-H) equations, we expect the consequences of this change will be entirely localized to the region of the membrane. As described by H-H (see reference 5 below) this ion flux will produce local changes in location and function of peripheral membrane proteins that constitute a rapid local response to the perturbation, so that information transmission to other cellular organelles is not necessary. This is similar to autonomic functions in multicellular organisms that deal with isolated, transient perturbations through local reflexes.

In contrast, we expect some environmental information will cause gate openings of multiple ion channels, and/or will maintain the channels in an open state for a longer period of time. This will increase the amplitude, time, and spatial distribution of changes in cytoplasmic ion concentrations. As we will see, it will also maximize the received information about the environment which, in turn, maximizes speed of signal flow and speed of decoding. We hypothesize that optimal cell function and survival will additionally necessitate that this information be transmitted to other organelles so as to elicit a more global cellular response. Examples are increased ATP production by the mitochondria and alterations in gene expression and translation.

In general, optimal communication in complex networks will integrate coarse- and fine-grained dynamics. Coarse-grained conduits transmit information from larger temporal and spatial scales to allow more rapid and efficient processing of large scale perturbations^30,31^. Fine-grained information dynamics focus upon the transmission of finer spatial and temporal scales^32^. Here, we hypothesize that local ion changes in the cytoplasm adjacent to open transmembrane channels can alter the terminal ends of local cytoskeletal structures. As shown in Figs 2 and 3, microtubules are relatively large^11^ (~30 nm in diameter) tubes with a hollow center. Empirical studies have demonstrated that signals in the form of ionic waves can be transmitted along the course of a microtubule^22^. We view information transmitted by the microtubule as “coarse grained^30^” because:

1. The microtubule is sufficiently large that it will primarily detect ion changes that occur within a cross sectional region similar to its diameter (~30 nm).
2. Most microtubules connect to the centrosome, which is typically positioned adjacent to the nuclear membrane with which it communicates^33^. While most recognized for its role in microtubule organization and spindle assembly, the centrosome it is also associated with molecules associated with diverse cellular functions including cell-cycle progression, checkpoint control, ubiquitin-mediated degradation^33,34^ and protein kinase A (PKA)^35^ which has diverse regulatory functions in cell metabolism^36^. Thus, the centrosome, in effect, will tend to “average” signals from multiple regions over time and communicates this summation to the nucleus.
3. The microtubule has a number of additional electro-magnetic^37,38^ properties that could additionally integrate the activities of multiple microtubules within the cell cytoplasm or even extend into the extracellular matrix or adjacent cells.

In contrast, microfilaments (diameter ~4 nm) can communicate in fine detail^39^. Prior studies have demonstrated that microfilaments, which are composed of actin with highly negative surface charge, are highly conductive and, in fact, have been used as nanowires that respond to osmotic and electric potential differences^18,22,38,40,41^. Because of its small diameter, we propose microfilaments, in contrast to microtubules, will transmit fine-grained information showing fluctuations on the order of microseconds (“fine-grained”). Hence we propose that changes in cytoplasmic ion concentrations near the membrane end of the microfilament–with the other end continuing to have the usual ion concentration distribution–will generate a potential difference across the microfilament **“**wire,**”** resulting in ion flow. Microfilaments typically attach to multi-protein complexes in the nuclear membrane which have been shown to control both gene expression and chromosomal locations. Thus, in this component of the system, signal transmission is both very rapid–“tunable” (in the sense that the electron flow is dependent on the potential along the length of the microtubule)–and well-resolved both spatially and temporally at both signal transmission and reception.

In summary, we propose that elements of the cytoskeleton mediate biomechanical activity and can likewise carry information. In addition, both microtubules and microfilaments connect with mitochondria and the endoplasmic reticulum, and can course along the cytoplasm adjacent to the cell membrane. This allows a broad distribution of information signals to enter many regions of the cell (and even flow to other cells). However, for simplicity, we here focus on information transmission between specifically the cell membrane and the nuclear membrane.

## Principles of Information Transmission

The dynamics governing information transmission have been extensively investigated by the pioneering work of Fisher, Shannon and others (see below). Ideal information transmission occurs when the receiver obtains precisely the information that was sent from its source. This is because, any channel carrying a signal from a sender to a receiver cannot (by definition of a channel) convey more information than is contained in the source signal. Instead, there is inevitable loss of source Shannon information en route during the process of encoding, transmission, reception and decoding of the message. Therefore, minimizing such loss is the realistic goal of such a system.

Note that this ignores the evolutionary cost to the system of acquiring the message. Instead, it tacitly assumes every such possible message to be acquired with equal cost, and focuses upon the issue of how well the system can respond to *that* message, regardless of cost. The assumption is that, *realistically*, *the external system activities producing the message are not controllable by the observer*. Hence, from the point of view of survival, evolutionary dynamics will optimize an organism’s response to any signal that can affect its fitness. This requires a maximum likelihood estimation^42,43^ approach rather than, e.g., one seeking a posterior mean, since the latter would require knowing the probability of each such possible message.

Meeting this goal of maximum likelihood estimation of external state properties in living systems requires: (a) minimization of the occurrence of data errors and (as below) (b) maximization of the rate of received information. Such a scenario is very beneficial for purposes of biological survival in the random, and possibly hostile, environment we are assuming. As will be seen, ion transmission through microtubules or microfilaments achieves this dual aim (a), (b).

As found below, coarse grained and fine grained information acquisition are subject to two different principles of minimum information loss (i) *I* − *J* = minimum and (ii)*S*_*I*_ − *S*_*J*_ = minimum, where subscripts *I* and *J* refer, respectively, to information received and at the environmental source. In this paper we focus on the environmental information that enters the cell in the form of a transmembrane flow of one or more ions. How well can that information be received? Also, can that information be transmitted optimally (as above) to other regions of the cell via the cytoskeleton network?

In principle (i), *I* and *J* are, respectively, levels of temporal Fisher information when just received and just sent, over the continuous, total time interval (0, *T*) of flow; likewise in principle (ii) for the Shannon informations *S*_*I*_ and *S*_*J*_. Then, by either principle *the information loss is minimized*. The particular choice of principle, (i) or (ii), is governed by the fineness of the spatial structure forming the information conduit, as follows.

In systems with true fine structure (order of 1–5 nm) information *I* is the ion’s level of Fisher information^44^, with *J* the equivalent physical information^45^. Depending on case, *J* could be the sum of all ion concentrations, mean times within the system; etc., affecting *I*. Or it could be the information as represented in a conjugate space to *t*, such as energy-momentum in quantum-relativistic problems^46–48^.

Thus, principle (i), *I*−*J* = minimum, operates on the finest level of cellular structure. It has been termed^46^ EPI (“extreme physical information”) and applies to the finest ion signal flow, through microfilaments of actin. Operating on this finest scale allows principle (i) to even give rise to quantum effects^45^, such as the Schrodinger wave equation (where, in particular, *J* is the mean kinetic energy^47^; although this is not the case here). But in all cases, *J* in principle (i) is the largest possible value of *I*. It results that principle (i) produces a maximized value for the Fisher information Principle (i) is shown later to give rise to the well-known Hodgkin-Huxley equations.

By comparison, principle (ii)*S*_*I*_ − *S*_*J*_ = minimum, where *S*_*I*_ and *S*_*J*_ are, respectively, the levels of Shannon information (in bits) as, respectively, received and sent. This applies when the system signals are coarse grained. It was first applied to telephone communication (*real* flow of electron charges through *real*, metal wires, represented by microfilaments of actin here), by C.E. Shannon^49^. For such coarse-grained microtubules (order of 25–100 nm) principle (ii) becomes ΔS ≡ *S*_*I*_ − *S*_*J*_ = *minimum*, one of *minimum loss* of Shannon information (ii). Principle (ii) is, thus, a non-quantum, coarse-grained theory. It results in the highest possible *delivered* information from an arbitrary source message in the environment. In fact principle (ii) *directly derives* as a coarse-grained version of principle (i) (see below). As a verification, one solution to problem (ii) of coarser ion flow is found to obey the classical Hodgkin-Huxley equations^6^ (see Eq. 5, below).

These calculations based on minimum information-loss principles (i) and (ii) indicate that real biological systems, such as the neuron of the giant squid obey ion flow rates delivering optimum levels of acquired information^3^. However, this ignores the issue of how the cells *use* information in the decoded messages to optimize survival and fitness^2^. To answer it, the benefits of acquiring and communicating *each component of available information of threat or benefit in the environment* would have to be weighed against the resources needed to maintain the molecular machinery necessary to cope with it. To do such a calculation would require knowing unknowable probabilities of unknown possible threats. This is, again, why we can only calculate the response to an arbitrary message of threat or benefit as in the preceding section.

Ideally, to maintain critically important cell functions loss of information in transmission must be *minimized* using a fine grain network. Analogously, a person translating a book from ancient hieroglyphics to English does not have to do it perfectly (i.e. with zero error) to produce a generally useful translation for consumption by the general public. However, translation of information regarding, for example, dates and places may be essential for historians or archaeologists. Here, the translation must be as close to perfect as possible (i.e. with minimum error).

Since principle (ii) of minimum loss of Shannon information *derives* (in Sec. 5) as a coarse-grained approximation to the EPI principle (i), and since all eukaryotes contain tubulin, probably all have likewise *evolved* out of the principle of minimum loss of Shannon information. As will be seen this is a necessary condition for natural selection.

In summary, we present a variational principle of biophysics that governs intracellular information flow based upon the granularity of the conduit through which the signal flows. We propose that the principle of minimum loss of Fisher information applies when fine grained information is transmitted electron flow through microfilaments (sometimes called ‘actin wires^16^’). In contrast, the principle of minimum loss of Shannon information during transmission governs coarse grained information carried by ion flow through microtubules. Importantly, however, we note that “minimum loss” also means, in a positive sense, *maximum gain* and thus can have the effect of increasing fitness. As we noted, these metrics of information transmission are directly related: When Fisher information undergoes coarse graining, it becomes proportional to Shannon information. This is, then, an important bridge between the discrete and continuous aspects of living systems.

## Fisher Information

All information forms used in this paper ultimately arise out of Fisher information. By definition, this obeys^44,45,47,48,50^:

1 $$I=4\,\int dt\,{[\frac{da}{dt}]}^{2},\,a=a(t)\equiv \sqrt{\,p(t)},$$

where *p* = *p*(*t*) is the probability density on position *t* for the ion and *a*(*t*) is defined as its (real) amplitude. All integrals are over a fixed time interval 0 ≤ *t* ≤ *T* of observation.For now, we notice that the form of Eq. (1) is also that of a Lagrangian *L* in integral $\int dtL$, and this is conventionally varied as δ$\int dtL=0$ to derive^45^ the quantum mechanics obeyed by amplitude law *a*(*t*) in scenarios of fine structure 1–5 nm or, alternatively, by classical flow of *p*(*t*) in coarser structure of size 25–100 nm. The emphasis in this paper is on the latter (classical domain) behavior.

The information *I* defined by Eq. (1) is also conventionally used to measure, by the relation^44,45,48^

2 $${e}_{min}^{2}=1/I,$$

the minimum possible mean-squared error *e*^2^ of any estimate of the true time *t*_0_ based on its repeated measurement *t*_*n*_ = *t*_0_ + *error*_*i*_ *n* = *1*, *… N*. Thus, *I* has the significance of defining how well a quantity on the continuum (here of time values *t*) *can be known*. (Notice that In Eq. (2), the larger information *I* is the smaller is the *rms* error *e*_*min*_, as one would expect of an information measure). Relation (2) has been the basis for usual *past* uses of Fisher information *I*.

By comparison, over the recent two decades another, completely different use of information *I* has arisen. Its aim is, not to merely measure particular values *t*_*n*_ of an observable phenomenon, as above, but rather to estimate the *actual probability law p*(*t*) governing *t* in the unknown phenomenon (of physics^47^, econophysics, biology^51^, cancer^52^ growth, chemistry, etc.^45,53^). The present paper extends these calculations to problems of ion- or electron transmission using principles (i) or (ii).

## Its Physical Manifestation *J*, EPI Principle

This is by using a principle of extreme “physical information” *I* − *J*,

3 $$I-J={\rm{minimum}}$$

through variation of *p*(*t*). Although both *I* and *J* are metrics of information hey differ in meaning. From the factor ${(\frac{da}{dt})}^{2}$ in Eq. (1), information *I* governs the amount of ‘slope’ or ‘roughness’ in both probability law *p*(*t*) and its amplitude law *a*(*t*). Also, by Eq. (2), *I* governs how accurately an unknown coordinate *t* can be known. The other quantity *J* in (3) defines the *meaning* of the information *I* as a *physical quantity*. Their difference *I−J* is called the ‘physical information,’ since it measures how much net information *I−J* contributes to the physical effect. Hence Eq. (3) expresses a principle of extreme net physical information (EPI). In our cellular scenario it, in fact, represents a scenario of minimum lost, temporal Fisher information. What does this mean?

Quantity *I−J* is always convex, so it defines a *minimum* value when varied mathematically. Such minimization means *I*≈*J*, i.e. the theoretical information tends to equal its physical manifestation. In fact, in *quantum* scenarios^45,48^ *I* = *J*, meaning the entire physical manifestation *J* of information *I* (here the energy) goes into forming the observable information *I*.

Meanwhile, in our case of cellular information flow *J* is input as the mean time of ion flow from the cytoplasm adjacent to the cell membrane to its other end at the nuclear membrane. This choice of *J* was that of Hodgkin and Huxley^6^. Hence, principle (3) provides the solution for amplitudes *a* = *a*(*t*) in signal transmission of fine structured time scale. The principle (3) of Extreme Physical Information or EPI has been used to derive most of textbook physics^46^ and some laws of biology^54^ including a prediction of power law growth $m(t)=m(0){t}^{\O }$ for early-growth stages of breast cancer^52,55^ where the constant $\O =1.618034$ that has been confirmed in multiple studies using mammography data.

## Transition to Principle of Minimum Kullback-Leibler (KL) Divergence

We demonstrate above that the Fisher information-based EPI principle (3) is directly applicable to problems of unknown ion rate functions *p*(*t*) or *a*(*t*) on the continuum of *t*, typified by spatial observations on the nanometer (fine) scale 1–10 nm (case of microfilaments). For this scale of problem the information *I* was found to be Fisher’s, given by Eq. (1).

However, as noted above, information transmission *via microtubules* instead requires the ions to flow along a much larger structure (~30 nm in diameter). This is a coarse-grained problem and our goal is to find the ‘coarse-grained’ rates *p*(*t*), *q*(*t*). To do this, we examine a transition from the fine-scaled principle (3) to the corresponding coarse-grained problem.

We start by using identity *p*(*t*) = *a*^2^(*t*) to express Eq. (1) it in terms of the probabilities *p*(*t*),

4 $$I=\int dt\frac{{(dp/dt)}^{2}}{p}.$$

However, for application of the EPI principle (3) to this discrete problem of the soma, we need the form of the information (4) where differentials *dt* are regarded as small but finite, “granular” changes Δ*t*. The latter are defined as follows.

In principle the finest time interval *dt* in principle (4) is of size zero. However, in practice it is the finite time interval Δ*t* during which the ion is located within the cell membrane. This is time between the instant that the ion just enters the cell membrane, from the outside environment, to the time that it just emerges from the cell membrane and enters the cell cytoplasm. On this basis, how large an information value *I* is delivered by the ion *i* to the observer during this time interval? Let it obey probability *q*_*i*_(*t*) in the outside environment. Then probability *p*_*i*_(*t*) after entering the cytoplasm a short time Δt later is *p*_*i*_(*t* + t). Then the information *I* in Eq. (4) is easily found to approximately obey^45^:

5 $${I}=2/({\rm{\Delta }}{{t}}^{2}){\int }_{0}^{\infty }\,dt{p}_{i}(t)\mathrm{ln}({p}_{i}(t)/{q}_{i}(t))\equiv {H}_{KL}({p}_{i}||{q}_{i})=minimum,\,\,i=\,1,\ldots ,N.$$

This is the information delivered to the observer as limited by the granular nature of the ion and the medium (cell membrane) it passes through. Notation *H*_*KL*_(*p*_*i*_||*q*_*i*_) denotes the Kullback-Leibler (K-L) divergence^53,56^ (or ‘distance’) between probabilities *p*_*i*_(*t*) and *q*_*i*_(*t*) (these are, equivalently, ion flow rates, since the random variable is the time *t*).

Factor 2/(Δ*t*^2^) in (5) shows that observing the time with a finer (smaller) “grain size” Δ*t* gives greater information *I* in cell structure such as a microtubule. This is intuitively correct. More importantly, Eq. (5) also generally represents *the loss in Shannon information*^53^ for an ion *i* passing through the membrane regarded as an information channel. By (5) the loss is, then, explicitly, minimized in this coarse-grained scenario. In summary, the information *I* is identically the loss in Shannon information^45^ during the flow from along the microtubule, *and this loss is minimized*. This is central to the information-based approach here. In summary, both principles (i) and (ii) define scenarios of *minimum loss of information*, although of different types – Fisher information in (i), and Shannon information in (ii).

Finally, even if grain size Δ*t* is not very small, by the approximate nature of principle (5) it may be used as simply a first-order (in change Δ*t*) approximation to the general principle (3). That is, a Shannon information-based calculation (ii) is always at least an approximate solution to the Fisher-information-based one (i).

## Insertion of Prior Knowledge

The minimum in Eq. (3) is to be obtained in the presence of prior knowledge *J* about the trajectories. These must, e.g., obey normalization. But the key prior knowledge was found by Hodgkin and Huxley^6^ to be the mean times *τ*_*i*_, *i* = 1, …, *N* for ions in the system (cochlea). The minimum value in Eq. (5) is also constrained by this knowledge. Using these as additive Lagrange constraints *J* during minimization of *I*, principle (5) becomes one of constrained *KL* (Kullback-Leibler) divergence^53^

6 $$\begin{array}{c}I-J={\int }_{0}^{\infty }\,dt{p}_{i}(t)\,\mathrm{ln}({p}_{i}(t)/{q}_{i}(t))+{\lambda }_{1}\int dt\,[{p}_{i}(t)-1]\\ \,\,+{\lambda }_{2}\int dt[{q}_{i}(t)-1]+{\lambda }_{3}[\int dtt{p}_{i}(t)-{\tau }_{i}]=min\end{array}$$

The first right-hand term is the *KL* divergence in (5). The terms in *λ*_1_ and *λ*_2_ express normalization of probability densities *p*_*i*_(*t*) and *q*_*i*_(*t*) or, more generally, the total presence of ion *i* over time interval (0, T). Notice that Eq. (6) is of the same general form as the EPI principle (3) and, hence, is called the *KL*_min_ principle. It has been found that this *KL*_min_ principle derives the equations governing the *actual* ion trajectories *p*_*i*_(*t*), *i* = 1, …, *N*, i.e. the Hodgkin-Huxley equations^6^. Further significance is that, because of the required minimum value in principle (5) the Shannon information is maximized for the transit of each ion *i* (i.e. its information *loss* is *minimized*).

Although, as was noted, this theory holds for transmembrane ion flows carrying a propagating signal along neurons, it holds as well for ion flux within microtubules.

## Discussion

Transmission of information from the cell membrane to the nucleus and other central organelles following ligand binding to membrane receptors has been extensively studied. Typically, the receptor binding triggers (often through intermediate proteins) phosphorylation of messenger proteins in the cytoplasm that then travel to other organelles. These information pathways are widely investigated and play important roles in cellular function as well as cancer development. However, we note that signal transmission via 3 dimensional random walk (notably, not obeying principle (5) of maximum acquired information level) inevitably suffers significant degradation of information regarding the time and location (on the cell membrane) of the perturbation^57^.

Under many circumstances, this lossy information is, nevertheless, sufficient to elicit a necessary response^58,59^ and has the benefit of low energy consumption^60^. However, acquiring even more information about the location and time of perturbation may be essential under some circumstances such as locating a predator or a potential food source by single cell eukaryotes, or moving to a correct cellular location within the highly ordered 3 dimensional structure of tissue in a multicellular organism. In prior work, we have proposed that eukaryotes use the difference in ion concentrations in the extra-cellular and intra-cellular fluid as a mechanism to receive, process, and respond to a wide range of perturbations in the environment. Indeed, the value of maintaining this membrane information receiver is evident in studies that show about 40% of the energy budget in eukaryotic cells^61^ is consumed by the ATP-dependent membrane ion pumps that maintain the gradient.

We have proposed cellular information dynamics include ion-specific transmembrane channels which permit communication in the form of ion flows between the environment and the cell. This occurs when the specialized gate (there are well over a hundred different types of gates) is induced to open by the environmental perturbation. Thus, the ion flow *in toto* actually represents an *optimized* response (5) to the nature of the perturbation, as well as its time and place. The subsequent local processing and response are described above.

In experimental studies of a highly specialized application of this principle – the ion flow carrying a traveling wave in neurons – it is clear that the change in local ion concentrations is rapidly dissipated through diffusion from adjacent regions of the cytoplasm and activation of the transmembrane ion pumps. However, in normal cell function, we anticipate that this information, although transient and spatially localized, will in many situations need to be communicated to other components of the cell.

Here we focus on the potential role of microfilaments and microtubules in this communication network. These long linear polymers are often arrayed in organized patterns and frequently observed to be oriented along the radius of the cell from the nuclear membrane to the cell membrane. The potential for both microtubules and microfilaments to transmit signals via ion conduction has been extensively investigated both theoretically and experimentally. Here we propose that extracellular information that is received by specialized gates in membrane ion channels and transmitted through ion transmembrane ion fluxes can be propagated by microfilaments and microtubules to the nucleus and other internal organelles such as the mitochondria. The specific properties of microfilaments and microtubules allow them to carry fine-grain or coarse-grain information respectively. In the case of microtubules, which typically converge on the centrosome, this coarse grain information allows rapid assessment of the overall state of the environment over time. Or in the case of microfilaments, which typically link to protein complexes on the nuclear membrane, the fine-grain information can convey, to the nucleus, detailed information about the spatial and temporal variations of the environment. In prior work^3,66,67^ we found these information dynamics to be highly optimized.

We note that such optimization, obeying information maximization principles (i) or (ii), give rise to optimally *fast and effective* responses to environmental challenges and benefits. Hence, these are a *necessary condition* for natural selection to have taken place (and continue).

Finally, we note that our analysis ignores possible communication between individual microfilaments and microtubules. In reality, microfilaments and microtubules frequently interface through direct physical contact and cross-linking proteins. It is also likely that the elements of the cytoskeleton can interact in complex way with molecular transduction pathways. This suggests a complex network for signal transmission and analysis that permits rich information dynamics that likely augments and modifies the more well-known and studied information found  molecular pathways (e.g. the MAPK pathway) that carry information following ligand binding to a membrane receptor to the nucelus.
