# Publisher Correction: Signal transmission through elements of the cytoskeleton form an optimized information network in eukaryotic cells

**DOI:** 10.1038/s41598-019-51083-2

**Published:** 2019-10-08

**Authors:** B. R. Frieden, R. A. Gatenby

**Affiliations:** 1 College of Optical Science, University of Arizona, Tucson, AZ USA; 2 Department of Integrated Mathematical Biology, Moffitt Cancer Center, Tampa, FL USA

Correction to: *Scientific Reports* 10.1038/s41598-019-42343-2, published online 16 April 2019

This Article contains errors.

In the section called ‘Its Physical Manifestation J, EPI Principle’,

“The principle (3) of Extreme Physical Information or EPI has been used to derive most of textbook physics^46^ and some laws of biology^54^ including a prediction of power law growth *m(t)* = *m(0)t*^*\O*^ for early-growth stages of breast cancer^52,55^ where the constant\O = 1.618034 that has been confirmed in multiple studies using mammography data.”

should read:

“The principle (3) of Extreme Physical Information or EPI has been used to derive most of textbook physics^46^ and some laws of biology^54^ including a prediction of power law growth *m*(*t*) = *m*(0)*t* ^ϕ^ for early-growth stages of breast cancer^52,55^ where the constant ϕ = 1.618034 is the Fibonacci golden mean, widely observed in biological growth processes including multiple studies of mammographic cancer data.”

In addition, in the section called ‘Transition to Principle of Minimum Kullback-Leibler (KL) Divergence’,

“Then probability ${p}_{i}(t)$ after entering the cytoplasm a short time $\Delta t$ later is ${p}_{i}(t+t)$.”

should read:

“Then probability is ${p}_{i}(t)$ after entering the cytoplasm a short time $\Delta t$ later is ${p}_{i}(t+\Delta t)$.”

Finally, Reference 45 which was incorrectly given as:

Frieden, B. R. & Frieden, B. R. *Science from Fisher information: a unification*. 2nd edn, (Cambridge University Press, 2004).

The correct reference is written below as Reference 1:

## References

[1] Frieden, B. R. *Science from Fisher information: a unification*. 2nd edn, (Cambridge University Press, 2004).

